# 5-Fluoro-*N*′-(4-methyl­cyclo­hexyl­idene)-3-phenyl-1*H*-indole-2-carbohydrazide

**DOI:** 10.1107/S1600536813018436

**Published:** 2013-07-06

**Authors:** Sevim Türktekin Çelikesir, Mehmet Akkurt, Gökçe Cihan Üstündağ, Gültaze Çapan, Orhan Büyükgüngör

**Affiliations:** a Department of Physics, Faculty of Sciences, Erciyes University, 38039 Kayseri, Turkey; b Department of Pharmaceutical Chemistry, Faculty of Pharmacy, Istanbul University, 34116 Beyazit, Istanbul, Turkey; c Department of Physics, Faculty of Arts and Sciences, Ondokuz Mayıs University, 55139 Samsun, Turkey

## Abstract

The title compound, C_22_H_22_FN_3_O, crystallized with two independent mol­ecules (*A* and *B*) in the asymmetric unit; these are linked by a pair of N—H⋯O hydrogen bonds, forming a pseudo-centrosymmetric dimer with an *R* ^2^_2_(10) motif. In addition, a number of C—H⋯π inter­actions are also observed. The 1*H*-indole ring systems in mol­ecules *A* and *B* are essentially planar [maximum deviations of 0.019 (2) and 0.014 (2) Å, respectively] and make dihedral angles of 77.64 (10) and 69.50 (9)°, respectively, with thephenyl rings.

## Related literature

For the synthesis and characterization of some bioactive indole derivatives, see: Akkurt *et al.* (2010 ▶, 2013 ▶); Cihan-Üstündağ & Çapan (2012 ▶); Zhang *et al.* (2004 ▶). For puckering analysis, see: Cremer & Pople (1975 ▶). For the graph-set analysis of hydrogen bonding, see: Bernstein *et al.* (1995 ▶).
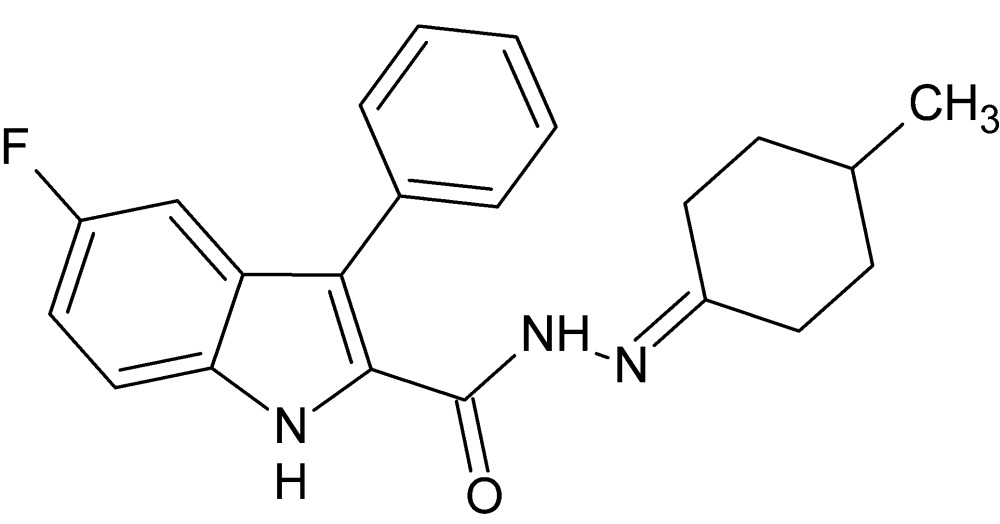


## Experimental

### 

#### Crystal data

C_22_H_22_FN_3_O
*M* *_r_* = 363.43
Triclinic, 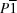

*a* = 11.6630 (6) Å
*b* = 13.5320 (7) Å
*c* = 14.7754 (8) Å
α = 112.967 (4)°
β = 95.936 (4)°
γ = 111.385 (4)°
*V* = 1915.4 (2) Å^3^
*Z* = 4
Mo *K*α radiation
μ = 0.09 mm^−1^
*T* = 296 K
0.68 × 0.52 × 0.33 mm

#### Data collection

Stoe IPDS 2 diffractometer
Absorption correction: integration (*X-RED32*; Stoe & Cie, 2002 ▶) *T*_min_ = 0.948, *T*_max_ = 0.972
26097 measured reflections
8702 independent reflections
5714 reflections with *I* > 2σ(*I*)
*R*_int_ = 0.058

#### Refinement

*R*[*F* ^2^ > 2σ(*F* ^2^)] = 0.057
*wR*(*F* ^2^) = 0.144
*S* = 1.03
8702 reflections
496 parameters
2 restraints
H atoms treated by a mixture of independent and constrained refinement
Δρ_max_ = 0.42 e Å^−3^
Δρ_min_ = −0.37 e Å^−3^

### 

Data collection: *X-AREA* (Stoe & Cie, 2002 ▶); cell refinement: *X-AREA*; data reduction: *X-RED32* (Stoe & Cie, 2002 ▶); program(s) used to solve structure: *SHELXS97* (Sheldrick, 2008 ▶); program(s) used to refine structure: *SHELXL97* (Sheldrick, 2008 ▶); molecular graphics: *ORTEP-3 for Windows* (Farrugia, 2012 ▶); software used to prepare material for publication: *WinGX* (Farrugia, 2012 ▶).

## Supplementary Material

Crystal structure: contains datablock(s) global, I. DOI: 10.1107/S1600536813018436/sj5341sup1.cif

Structure factors: contains datablock(s) I. DOI: 10.1107/S1600536813018436/sj5341Isup2.hkl

Supplementary material file. DOI: 10.1107/S1600536813018436/sj5341Isup3.cml

Additional supplementary materials:  crystallographic information; 3D view; checkCIF report

## Figures and Tables

**Table 1:** Hydrogen-bond geometry (Å, °) *Cg*1, *Cg*2, *Cg*3, *Cg*6 and *Cg*8 are the centroids of the 1*H*-pyrrole and benzene rings of the 1*H*-indole ring system of mol­ecule *A*, the phenyl ring of mol­ecule *A*, the 1*H*-pyrrole ring of the 1*H*-indole ring system of mol­ecule *B* and the phenyl ring of mol­ecule *B*, respectively.

*D*—H⋯*A*	*D*—H	H⋯*A*	*D*⋯*A*	*D*—H⋯*A*
N1—H1⋯O2	0.86	2.15	2.895 (2)	145
N4—H4⋯O1	0.86	2.04	2.811 (2)	149
C17—H17*A*⋯*Cg*1^i^	0.97	2.66	3.594 (3)	163
C17—H17*B*⋯*Cg*3	0.97	2.74	3.685 (3)	164
C31—H31⋯*Cg*6^ii^	0.93	2.87	3.658 (2)	144
C35—H35⋯*Cg*2^iii^	0.93	2.96	3.627 (3)	130
C39—H39*B*⋯*Cg*8	0.97	2.72	3.667 (3)	164
C42—H42*A*⋯*Cg*1^iv^	0.97	2.99	3.848 (3)	148

Symmetry codes: (i) 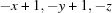; (ii) 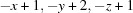; (iii) 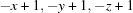; (iv) 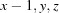.

**Fig. 1.**
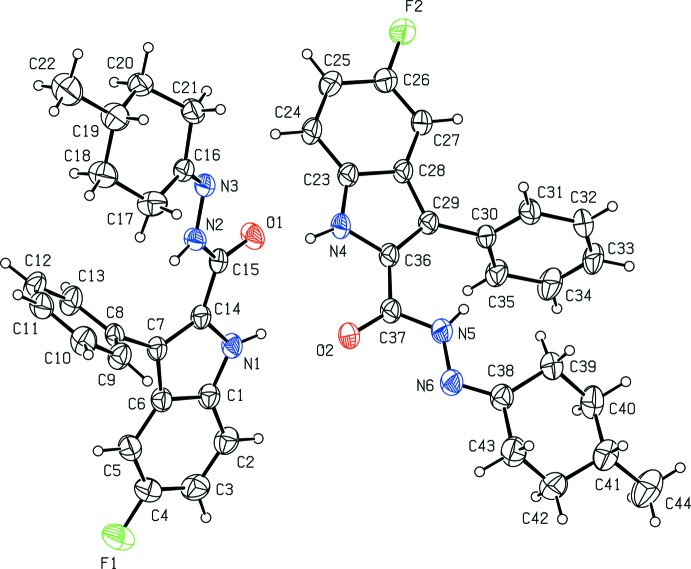
The molecular structure of (I), showing the atom labelling scheme. Displacement ellipsoids for non-H atoms are drawn at the 30% probability level.

**Fig. 2.**
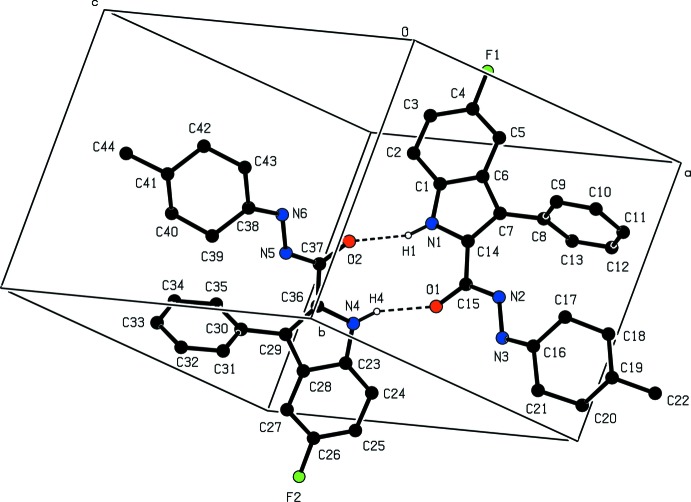
View of the N—H···O dimer in the unit cell. H atoms not participating in hydrogen bonding have been omitted for clarity and hydrogen bonds are drawn as dashed lines.

## supplementary crystallographic information

### Comment

Indole-2-carbohydrazides are attractive targets in organic synthesis because of the biological potential of the indole scaffold and the synthetic utility of the carbohydrazide function (Zhang *et al.*, 2004; Akkurt *et al.*, 2010; 2013). The title compound has been synthesized as a member of a series of indolylhydrazones with antituberculosis properties (Cihan-Üstündağ & Çapan, 2012). To fully characterize the structure, we now report on the X-ray diffraction analysis of the title compound.

In the title compound, (I), (Fig. 1), the asymmetric unit contains two crystallographically independent molecules, A and B, whose cyclohexane rings adopt distorted chair conformations [the puckering parameters (Cremer & Pople, 1975) are Q_T_ = 0.520 (3) Å, θ = 168.2 (3)°, φ = 31.9 (15)° for molecule A (with N1), and Q_T_ = 0.520 (3) Å, θ = 168.2 (3)°, φ = 31.9 (15)° for molecule B (with N4)].

The 1*H*-indole ring systems of both molecules A and B are essentially planar [maximum deviations are 0.019 (2) Å for C1 in molecule A and 0.014 (2) Å for C26 in molecule B]. The 1*H*-indole ring systems of molecules A and B make dihedral angles of 77.64 (10) and 69.50 (9)° with their phenyl rings, respectively. The C14–C15–N2–N3, C15–N2–N3–C16, C36–C37–N5–N6, C37–N5–N6–C38 torsion angles are 174.92 (18), -175.2 (2), -179.95 (17) and 178.4 (2)°, respectively.

In the crystal, the two molecules in the asymmetric unit are connected to each other, forming N—H···O dimers (Table 1, Fig. 2), giving rise to *R*_2_^2^(10) ring patterns (Bernstein *et al.*, 1995). Furthermore, C—H···π interactions (Table 1) contribute to the stability of the crystal packing in (I).

### Experimental

A mixture of 5-fluoro-3-phenyl-1*H*-indole-2-carbohydrazide (0.005 mol) and 4-methyl cyclohexanone (0.007 mol) was refluxed in 15 ml *ABS*. ethanol for 5 h. The precipitate obtained was purified by recrystallization from an ethanol-water mixture.

Yield:73%, mp.: 466.5–468.5 K. IR(KBr): υ_max_ 3348, 3240 (N—H), 1652 (C=O) cm^-1. 1^H-NMR (DMSO-d_6_/500 MHz): δ 0.87 (d, 3H, J=5.3 Hz, 4-CH_3_-cyc.*), 1.00–1.14 (m, 1H, CH_2_-cyc.), 1.44–1.64 (br. m, 4H, CH, CH_2_-cyc.), 1.66–1.84 (m, 2H, CH_2_-cyc.), 2.15 (s, 1H, CH_2_-cyc.), 2.29 (s, 1H, CH_2_-cyc.), 7.12 (br. t, 2H, J=8.5 Hz, H4,H6-ind.), 7.33–7.50 (m, 6H, H7, 3-C_6_H_5_-ind.), 9.44 (s, 1H, CONH), 12.02 (s, 1H, NH) p.p.m.. Analysis calculated for C_22_H_22_FN_3_O: C 72.71, H 6.10, N 11.56%. Found: C 72.67, H 6.39, N 11.57%. (*cyc.=cyclohexylidene, ind.=indole).

### Refinement

H atoms bonded to C atoms and the H atoms (N1)H1 and (N4)H4 of the two of the four amide groups were positioned geometrically with C—H = 0.93 - 0.98 Å, and N—H =0.86 Å and refined using a riding model with *U*_iso_(H) = 1.2 or 1.5*U*_eq_(C,*N*). The H atoms (N2)H2N and (N5)H5N of the two amide groups were found in a difference Fourier map and were refined freely. The crystal studied was a non-merohedral twin (twin law 0.24 0.00 - 0.75 - 0.09 - 1.00 0.05 - 1.26 0.00 - 0.24), with the minor twin component refining to 0.00116 (8).

### Crystal data

**Table:**

C_22_H_22_FN_3_O	*Z* = 4
*M**_r_* = 363.43	*F*(000) = 768
Triclinic, *P*1	*D*_x_ = 1.260 Mg m^−^^3^
Hall symbol: -P 1	Mo *K*α radiation, λ = 0.71073 Å
*a* = 11.6630 (6) Å	Cell parameters from 35686 reflections
*b* = 13.5320 (7) Å	θ = 2.0–28.0°
*c* = 14.7754 (8) Å	µ = 0.09 mm^−^^1^
α = 112.967 (4)°	*T* = 296 K
β = 95.936 (4)°	Prism, colourless
γ = 111.385 (4)°	0.68 × 0.52 × 0.33 mm
*V* = 1915.4 (2) Å^3^	

### Data collection

**Table:**

Stoe IPDS 2 diffractometer	8702 independent reflections
Radiation source: sealed X-ray tube, 12 x 0.4 mm long-fine focus	5714 reflections with *I* > 2σ(*I*)
Plane graphite monochromator	*R*_int_ = 0.058
Detector resolution: 6.67 pixels mm^-1^	θ_max_ = 27.6°, θ_min_ = 2.0°
ω scans	*h* = −15→15
Absorption correction: integration (*X-RED32*; Stoe & Cie, 2002)	*k* = −17→17
*T*_min_ = 0.948, *T*_max_ = 0.972	*l* = −19→19
26097 measured reflections	

### Refinement

**Table:**

Refinement on *F*^2^	2 restraints
Least-squares matrix: full	Hydrogen site location: mixed
*R*[*F*^2^ > 2σ(*F*^2^)] = 0.057	H atoms treated by a mixture of independent and constrained refinement
*wR*(*F*^2^) = 0.144	W = 1/[Σ^2^(*F*O^2^) + (0.0744*P*)^2^ + 0.1246*P*] WHERE *P* = (*F*O^2^ + 2*F*C^2^)/3
*S* = 1.03	(Δ/σ)_max_ < 0.001
8702 reflections	Δρ_max_ = 0.42 e Å^−^^3^
496 parameters	Δρ_min_ = −0.37 e Å^−^^3^

### Special details

**Table:**

Geometry. Bond distances, angles *etc*. have been calculated using the rounded fractional coordinates. All su's are estimated from the variances of the (full) variance-covariance matrix. The cell e.s.d.'s are taken into account in the estimation of distances, angles and torsion angles
Refinement. Refinement on *F*^2^ for ALL reflections except those flagged by the user for potential systematic errors. Weighted *R*-factors *wR* and all goodnesses of fit *S* are based on *F*^2^, conventional *R*-factors *R* are based on *F*, with *F* set to zero for negative *F*^2^. The observed criterion of *F*^2^ > σ(*F*^2^) is used only for calculating -*R*-factor-obs *etc*. and is not relevant to the choice of reflections for refinement. *R*-factors based on *F*^2^ are statistically about twice as large as those based on *F*, and *R*-factors based on ALL data will be even larger.

### Fractional atomic coordinates and isotropic or equivalent isotropic displacement parameters (Å^2^)

**Table:**

	*x*	*y*	*z*	*U*_iso_*/*U*_eq_	
F1	0.33494 (17)	−0.02996 (12)	0.06230 (14)	0.1113 (7)	
O1	0.68124 (14)	0.68746 (12)	0.28626 (9)	0.0679 (5)	
N1	0.52435 (14)	0.45358 (13)	0.24287 (11)	0.0543 (5)	
N2	0.70784 (16)	0.62496 (14)	0.12738 (12)	0.0547 (5)	
N3	0.78349 (15)	0.73959 (13)	0.14306 (11)	0.0564 (5)	
C1	0.46778 (18)	0.33259 (17)	0.20839 (13)	0.0553 (6)	
C2	0.3836 (2)	0.2633 (2)	0.24439 (16)	0.0669 (7)	
C3	0.3400 (2)	0.1422 (2)	0.19316 (18)	0.0771 (9)	
C4	0.3792 (2)	0.09106 (19)	0.10798 (19)	0.0761 (8)	
C5	0.4602 (2)	0.15512 (18)	0.06997 (16)	0.0678 (7)	
C6	0.50520 (18)	0.27986 (16)	0.12087 (13)	0.0547 (6)	
C7	0.58613 (17)	0.37471 (16)	0.10276 (12)	0.0510 (5)	
C8	0.64165 (17)	0.35989 (15)	0.01471 (12)	0.0495 (5)	
C9	0.56439 (19)	0.32276 (19)	−0.08081 (14)	0.0640 (7)	
C10	0.6149 (2)	0.3174 (2)	−0.16231 (14)	0.0724 (8)	
C11	0.7423 (2)	0.34579 (18)	−0.15024 (14)	0.0685 (7)	
C12	0.8187 (2)	0.3774 (2)	−0.05808 (16)	0.0773 (8)	
C13	0.7693 (2)	0.3848 (2)	0.02451 (14)	0.0689 (7)	
C14	0.59506 (17)	0.47922 (16)	0.17903 (12)	0.0501 (5)	
C15	0.66499 (16)	0.60585 (16)	0.20329 (12)	0.0499 (5)	
C16	0.81127 (17)	0.74851 (16)	0.06423 (14)	0.0546 (6)	
C17	0.7672 (2)	0.6516 (2)	−0.04384 (15)	0.0716 (7)	
C18	0.8715 (3)	0.66082 (19)	−0.09684 (16)	0.0813 (8)	
C19	0.9486 (3)	0.7848 (2)	−0.08349 (18)	0.0837 (9)	
C20	1.0026 (2)	0.87295 (18)	0.02981 (17)	0.0725 (7)	
C21	0.8962 (2)	0.87190 (18)	0.08106 (17)	0.0705 (8)	
C22	1.0472 (3)	0.7915 (3)	−0.1398 (3)	0.1207 (14)	
F2	0.95169 (12)	1.23386 (12)	0.77835 (10)	0.0893 (5)	
O2	0.39600 (13)	0.58695 (12)	0.35468 (10)	0.0678 (5)	
N4	0.62682 (15)	0.79327 (14)	0.47138 (11)	0.0576 (5)	
N5	0.28375 (16)	0.68606 (16)	0.42914 (13)	0.0627 (6)	
N6	0.16405 (16)	0.59508 (15)	0.36881 (11)	0.0648 (5)	
C23	0.72321 (18)	0.90083 (16)	0.54295 (13)	0.0535 (6)	
C24	0.85522 (19)	0.95099 (19)	0.55570 (15)	0.0632 (7)	
C25	0.9302 (2)	1.0619 (2)	0.63591 (16)	0.0674 (7)	
C26	0.8732 (2)	1.12177 (18)	0.70176 (15)	0.0645 (7)	
C27	0.74541 (18)	1.07482 (17)	0.69316 (14)	0.0589 (6)	
C28	0.66725 (17)	0.96119 (15)	0.61105 (12)	0.0506 (6)	
C29	0.53239 (17)	0.88540 (15)	0.57767 (12)	0.0501 (6)	
C30	0.43961 (16)	0.91430 (15)	0.63207 (12)	0.0498 (5)	
C31	0.4100 (2)	1.00510 (18)	0.63397 (15)	0.0653 (7)	
C32	0.3272 (2)	1.0345 (2)	0.68837 (18)	0.0777 (8)	
C33	0.2746 (2)	0.9750 (2)	0.74062 (18)	0.0849 (9)	
C34	0.3030 (2)	0.8843 (2)	0.73919 (18)	0.0847 (10)	
C35	0.3847 (2)	0.85356 (19)	0.68489 (15)	0.0659 (7)	
C36	0.51112 (17)	0.78338 (16)	0.49088 (12)	0.0513 (6)	
C37	0.39356 (18)	0.67621 (16)	0.41905 (13)	0.0541 (6)	
C38	0.06794 (19)	0.61469 (18)	0.38764 (14)	0.0626 (6)	
C39	0.0688 (2)	0.7241 (2)	0.46963 (18)	0.0765 (8)	
C40	−0.0321 (2)	0.6933 (2)	0.52374 (18)	0.0854 (9)	
C41	−0.1642 (2)	0.6026 (2)	0.45358 (19)	0.0816 (10)	
C42	−0.1578 (2)	0.4916 (2)	0.3791 (2)	0.0907 (10)	
C43	−0.0627 (2)	0.5196 (2)	0.31960 (18)	0.0865 (9)	
C44	−0.2606 (3)	0.5777 (4)	0.5126 (3)	0.1196 (16)	
H1	0.51700	0.50530	0.29580	0.0650*	
H2	0.35810	0.29860	0.30130	0.0800*	
H2N	0.688 (2)	0.563 (2)	0.0781 (14)	0.068 (6)*	
H3	0.28380	0.09360	0.21520	0.0920*	
H5	0.48450	0.11810	0.01300	0.0810*	
H9	0.47700	0.30100	−0.09040	0.0770*	
H10	0.56190	0.29450	−0.22550	0.0870*	
H11	0.77660	0.34350	−0.20470	0.0820*	
H12	0.90490	0.39420	−0.05030	0.0930*	
H13	0.82280	0.40670	0.08710	0.0830*	
H17A	0.69710	0.65430	−0.08270	0.0860*	
H17B	0.73410	0.57470	−0.04360	0.0860*	
H18A	0.92920	0.63560	−0.07030	0.0980*	
H18B	0.83270	0.60620	−0.16950	0.0980*	
H19	0.88800	0.80630	−0.11310	0.1000*	
H20A	1.05170	0.95280	0.03810	0.0870*	
H20B	1.06030	0.85250	0.06290	0.0870*	
H21A	0.93390	0.92490	0.15410	0.0850*	
H21B	0.84470	0.90180	0.05370	0.0850*	
H22A	1.10930	0.77190	−0.11260	0.1450*	
H22B	1.08940	0.87120	−0.13210	0.1450*	
H22C	1.00660	0.73590	−0.21130	0.1450*	
H4	0.63670	0.73980	0.42170	0.0690*	
H5N	0.2992 (17)	0.750 (2)	0.4704 (16)	0.067 (6)*	
H24	0.89120	0.91000	0.51080	0.0760*	
H25	1.01860	1.09750	0.64670	0.0810*	
H27	0.71140	1.11640	0.73980	0.0710*	
H31	0.44570	1.04670	0.59870	0.0780*	
H32	0.30760	1.09540	0.68900	0.0930*	
H33	0.21960	0.99550	0.77720	0.1020*	
H34	0.26700	0.84340	0.77490	0.1020*	
H35	0.40290	0.79170	0.68390	0.0790*	
H39A	0.05350	0.77160	0.43910	0.0920*	
H39B	0.15300	0.77220	0.51960	0.0920*	
H40A	−0.00490	0.66310	0.56740	0.1020*	
H40B	−0.03670	0.76600	0.56770	0.1020*	
H41	−0.19180	0.63690	0.41320	0.0980*	
H42A	−0.24240	0.43520	0.33140	0.1090*	
H42B	−0.13290	0.45420	0.41660	0.1090*	
H43A	−0.05610	0.44730	0.27760	0.1040*	
H43B	−0.09490	0.54540	0.27410	0.1040*	
H44A	−0.26420	0.65070	0.55500	0.1440*	
H44B	−0.23490	0.54640	0.55490	0.1440*	
H44C	−0.34390	0.52030	0.46520	0.1440*	

### Atomic displacement parameters (Å^2^)

**Table:**

	*U*^11^	*U*^22^	*U*^33^	*U*^12^	*U*^13^	*U*^23^
F1	0.1200 (13)	0.0613 (8)	0.1456 (14)	0.0303 (9)	0.0534 (11)	0.0465 (9)
O1	0.0915 (10)	0.0593 (8)	0.0553 (7)	0.0372 (8)	0.0418 (7)	0.0201 (6)
N1	0.0650 (10)	0.0590 (9)	0.0493 (7)	0.0337 (8)	0.0316 (7)	0.0251 (7)
N2	0.0649 (10)	0.0471 (8)	0.0495 (8)	0.0234 (8)	0.0289 (7)	0.0182 (7)
N3	0.0590 (9)	0.0512 (8)	0.0618 (8)	0.0266 (8)	0.0326 (7)	0.0230 (7)
C1	0.0599 (11)	0.0630 (11)	0.0557 (9)	0.0329 (10)	0.0258 (8)	0.0317 (9)
C2	0.0704 (13)	0.0824 (14)	0.0676 (11)	0.0383 (12)	0.0347 (10)	0.0452 (11)
C3	0.0762 (15)	0.0790 (15)	0.0914 (15)	0.0298 (13)	0.0344 (12)	0.0554 (13)
C4	0.0800 (15)	0.0568 (12)	0.0924 (15)	0.0273 (12)	0.0287 (12)	0.0370 (11)
C5	0.0749 (14)	0.0607 (12)	0.0730 (12)	0.0350 (11)	0.0308 (11)	0.0288 (10)
C6	0.0586 (11)	0.0575 (10)	0.0565 (9)	0.0314 (9)	0.0247 (8)	0.0270 (8)
C7	0.0552 (10)	0.0563 (10)	0.0493 (8)	0.0309 (9)	0.0241 (8)	0.0237 (8)
C8	0.0573 (10)	0.0481 (9)	0.0465 (8)	0.0288 (8)	0.0238 (8)	0.0177 (7)
C9	0.0580 (11)	0.0761 (13)	0.0534 (10)	0.0312 (11)	0.0201 (9)	0.0232 (9)
C10	0.0820 (15)	0.0837 (15)	0.0470 (9)	0.0384 (13)	0.0228 (10)	0.0234 (10)
C11	0.0844 (15)	0.0646 (12)	0.0539 (10)	0.0337 (11)	0.0386 (10)	0.0196 (9)
C12	0.0593 (12)	0.0933 (16)	0.0709 (13)	0.0368 (12)	0.0342 (11)	0.0236 (11)
C13	0.0608 (12)	0.0920 (15)	0.0512 (9)	0.0410 (12)	0.0212 (9)	0.0226 (10)
C14	0.0550 (10)	0.0583 (10)	0.0462 (8)	0.0304 (9)	0.0254 (7)	0.0252 (8)
C15	0.0528 (10)	0.0581 (10)	0.0469 (8)	0.0320 (9)	0.0251 (7)	0.0224 (8)
C16	0.0539 (10)	0.0545 (10)	0.0623 (10)	0.0273 (9)	0.0282 (8)	0.0275 (8)
C17	0.0747 (14)	0.0708 (13)	0.0549 (10)	0.0169 (11)	0.0197 (10)	0.0295 (10)
C18	0.1014 (18)	0.0663 (13)	0.0631 (12)	0.0262 (13)	0.0425 (12)	0.0231 (10)
C19	0.1068 (18)	0.0744 (14)	0.0788 (14)	0.0367 (14)	0.0553 (14)	0.0397 (12)
C20	0.0766 (14)	0.0552 (11)	0.0834 (13)	0.0224 (11)	0.0436 (12)	0.0310 (10)
C21	0.0832 (15)	0.0560 (11)	0.0819 (13)	0.0344 (11)	0.0445 (12)	0.0323 (10)
C22	0.166 (3)	0.0908 (19)	0.130 (2)	0.058 (2)	0.108 (2)	0.0568 (18)
F2	0.0637 (8)	0.0726 (8)	0.0934 (9)	0.0224 (7)	0.0195 (7)	0.0107 (7)
O2	0.0712 (9)	0.0612 (8)	0.0633 (7)	0.0334 (7)	0.0345 (7)	0.0141 (6)
N4	0.0643 (10)	0.0611 (9)	0.0542 (8)	0.0353 (8)	0.0347 (7)	0.0217 (7)
N5	0.0600 (10)	0.0581 (10)	0.0574 (9)	0.0282 (9)	0.0224 (8)	0.0115 (8)
N6	0.0615 (10)	0.0671 (10)	0.0511 (8)	0.0295 (9)	0.0185 (7)	0.0121 (7)
C23	0.0591 (11)	0.0580 (10)	0.0523 (9)	0.0303 (9)	0.0301 (8)	0.0262 (8)
C24	0.0627 (12)	0.0732 (13)	0.0670 (11)	0.0391 (11)	0.0382 (10)	0.0316 (10)
C25	0.0560 (12)	0.0743 (13)	0.0743 (12)	0.0296 (11)	0.0304 (10)	0.0330 (11)
C26	0.0613 (12)	0.0613 (12)	0.0624 (11)	0.0250 (10)	0.0218 (9)	0.0217 (9)
C27	0.0628 (12)	0.0591 (11)	0.0564 (9)	0.0312 (10)	0.0289 (9)	0.0215 (9)
C28	0.0562 (10)	0.0550 (10)	0.0517 (9)	0.0301 (9)	0.0297 (8)	0.0263 (8)
C29	0.0571 (11)	0.0533 (10)	0.0488 (8)	0.0282 (9)	0.0280 (8)	0.0252 (8)
C30	0.0526 (10)	0.0512 (9)	0.0438 (8)	0.0249 (8)	0.0232 (7)	0.0161 (7)
C31	0.0738 (13)	0.0611 (11)	0.0727 (12)	0.0370 (11)	0.0381 (10)	0.0310 (10)
C32	0.0784 (15)	0.0675 (13)	0.0924 (15)	0.0461 (12)	0.0401 (13)	0.0251 (12)
C33	0.0807 (15)	0.0965 (17)	0.0811 (14)	0.0510 (14)	0.0527 (13)	0.0262 (13)
C34	0.0865 (16)	0.1159 (19)	0.0835 (14)	0.0558 (15)	0.0595 (13)	0.0568 (14)
C35	0.0705 (13)	0.0786 (13)	0.0714 (12)	0.0408 (11)	0.0435 (10)	0.0429 (11)
C36	0.0591 (11)	0.0551 (10)	0.0486 (8)	0.0303 (9)	0.0306 (8)	0.0239 (8)
C37	0.0636 (11)	0.0583 (10)	0.0485 (8)	0.0317 (9)	0.0310 (8)	0.0241 (8)
C38	0.0644 (12)	0.0696 (12)	0.0514 (9)	0.0327 (11)	0.0206 (9)	0.0218 (9)
C39	0.0735 (14)	0.0701 (14)	0.0823 (14)	0.0390 (12)	0.0305 (12)	0.0231 (11)
C40	0.0867 (17)	0.1039 (18)	0.0774 (14)	0.0603 (16)	0.0352 (13)	0.0333 (13)
C41	0.0775 (16)	0.1055 (19)	0.0903 (15)	0.0522 (15)	0.0395 (13)	0.0573 (14)
C42	0.0626 (14)	0.0847 (17)	0.1170 (19)	0.0308 (13)	0.0188 (13)	0.0428 (15)
C43	0.0684 (15)	0.0888 (17)	0.0716 (13)	0.0378 (13)	0.0089 (11)	0.0084 (12)
C44	0.104 (2)	0.174 (3)	0.144 (3)	0.077 (2)	0.073 (2)	0.109 (3)

### Geometric parameters (Å, º)

**Table:**

F1—C4	1.361 (3)	C18—H18B	0.9700
F2—C26	1.361 (3)	C19—H19	0.9800
O1—C15	1.225 (2)	C20—H20B	0.9700
O2—C37	1.224 (2)	C20—H20A	0.9700
N1—C1	1.366 (3)	C21—H21B	0.9700
N1—C14	1.378 (3)	C21—H21A	0.9700
N2—C15	1.350 (3)	C22—H22A	0.9600
N2—N3	1.385 (3)	C22—H22B	0.9600
N3—C16	1.279 (3)	C22—H22C	0.9600
N1—H1	0.8600	C23—C24	1.395 (3)
N2—H2N	0.80 (2)	C23—C28	1.410 (3)
N4—C36	1.379 (3)	C24—C25	1.365 (3)
N4—C23	1.364 (3)	C25—C26	1.399 (3)
N5—N6	1.376 (3)	C26—C27	1.360 (3)
N5—C37	1.352 (3)	C27—C28	1.400 (3)
N6—C38	1.276 (3)	C28—C29	1.427 (3)
N4—H4	0.8600	C29—C30	1.488 (3)
N5—H5N	0.78 (2)	C29—C36	1.384 (3)
C1—C6	1.413 (3)	C30—C31	1.384 (3)
C1—C2	1.397 (3)	C30—C35	1.384 (3)
C2—C3	1.364 (4)	C31—C32	1.389 (3)
C3—C4	1.394 (3)	C32—C33	1.358 (4)
C4—C5	1.361 (4)	C33—C34	1.376 (4)
C5—C6	1.404 (3)	C34—C35	1.383 (3)
C6—C7	1.431 (3)	C36—C37	1.469 (3)
C7—C8	1.489 (3)	C38—C43	1.494 (3)
C7—C14	1.380 (3)	C38—C39	1.499 (3)
C8—C9	1.382 (3)	C39—C40	1.515 (4)
C8—C13	1.379 (3)	C40—C41	1.501 (4)
C9—C10	1.383 (3)	C41—C42	1.511 (4)
C10—C11	1.365 (4)	C41—C44	1.515 (5)
C11—C12	1.359 (3)	C42—C43	1.517 (4)
C12—C13	1.387 (3)	C24—H24	0.9300
C14—C15	1.473 (3)	C25—H25	0.9300
C16—C21	1.500 (3)	C27—H27	0.9300
C16—C17	1.496 (3)	C31—H31	0.9300
C17—C18	1.507 (4)	C32—H32	0.9300
C18—C19	1.506 (4)	C33—H33	0.9300
C19—C22	1.485 (5)	C34—H34	0.9300
C19—C20	1.516 (3)	C35—H35	0.9300
C20—C21	1.518 (3)	C39—H39A	0.9700
C2—H2	0.9300	C39—H39B	0.9700
C3—H3	0.9300	C40—H40A	0.9700
C5—H5	0.9300	C40—H40B	0.9700
C9—H9	0.9300	C41—H41	0.9800
C10—H10	0.9300	C42—H42A	0.9700
C11—H11	0.9300	C42—H42B	0.9700
C12—H12	0.9300	C43—H43A	0.9700
C13—H13	0.9300	C43—H43B	0.9700
C17—H17B	0.9700	C44—H44A	0.9600
C17—H17A	0.9700	C44—H44B	0.9600
C18—H18A	0.9700	C44—H44C	0.9600
			
C1—N1—C14	108.92 (16)	H21A—C21—H21B	108.00
N3—N2—C15	120.98 (16)	C19—C22—H22A	110.00
N2—N3—C16	115.91 (16)	C19—C22—H22B	109.00
C1—N1—H1	126.00	H22A—C22—H22C	110.00
C14—N1—H1	126.00	C19—C22—H22C	109.00
N3—N2—H2N	128.4 (19)	H22A—C22—H22B	109.00
C15—N2—H2N	110.5 (18)	H22B—C22—H22C	109.00
C23—N4—C36	109.59 (16)	N4—C23—C24	130.90 (19)
N6—N5—C37	122.72 (18)	N4—C23—C28	107.45 (19)
N5—N6—C38	116.56 (18)	C24—C23—C28	121.65 (18)
C36—N4—H4	125.00	C23—C24—C25	118.2 (2)
C23—N4—H4	125.00	C24—C25—C26	119.6 (2)
C37—N5—H5N	110.4 (17)	F2—C26—C27	118.9 (2)
N6—N5—H5N	126.8 (17)	C25—C26—C27	123.9 (2)
N1—C1—C6	107.95 (18)	F2—C26—C25	117.2 (2)
N1—C1—C2	130.44 (19)	C26—C27—C28	117.09 (19)
C2—C1—C6	121.6 (2)	C27—C28—C29	132.93 (19)
C1—C2—C3	117.7 (2)	C23—C28—C29	107.56 (16)
C2—C3—C4	120.3 (2)	C23—C28—C27	119.52 (19)
F1—C4—C3	117.2 (2)	C28—C29—C36	106.29 (18)
F1—C4—C5	118.9 (2)	C30—C29—C36	129.37 (19)
C3—C4—C5	123.9 (2)	C28—C29—C30	124.30 (16)
C4—C5—C6	116.8 (2)	C29—C30—C31	120.77 (18)
C5—C6—C7	133.18 (19)	C29—C30—C35	120.51 (19)
C1—C6—C5	119.7 (2)	C31—C30—C35	118.7 (2)
C1—C6—C7	107.12 (18)	C30—C31—C32	120.3 (2)
C6—C7—C8	125.71 (17)	C31—C32—C33	120.6 (2)
C8—C7—C14	127.95 (19)	C32—C33—C34	119.7 (2)
C6—C7—C14	106.25 (17)	C33—C34—C35	120.4 (2)
C7—C8—C13	122.73 (16)	C30—C35—C34	120.4 (2)
C9—C8—C13	117.73 (18)	N4—C36—C29	109.11 (17)
C7—C8—C9	119.52 (19)	N4—C36—C37	118.25 (16)
C8—C9—C10	121.1 (2)	C29—C36—C37	132.6 (2)
C9—C10—C11	120.20 (19)	N5—C37—C36	114.19 (18)
C10—C11—C12	119.6 (2)	O2—C37—N5	123.4 (2)
C11—C12—C13	120.6 (2)	O2—C37—C36	122.4 (2)
C8—C13—C12	120.69 (19)	N6—C38—C43	117.47 (19)
N1—C14—C7	109.76 (18)	C39—C38—C43	114.5 (2)
N1—C14—C15	117.64 (16)	N6—C38—C39	128.0 (2)
C7—C14—C15	132.59 (18)	C38—C39—C40	112.2 (2)
O1—C15—C14	121.94 (17)	C39—C40—C41	114.7 (2)
N2—C15—C14	115.28 (16)	C40—C41—C42	109.5 (2)
O1—C15—N2	122.8 (2)	C42—C41—C44	113.5 (3)
N3—C16—C21	116.31 (18)	C40—C41—C44	111.9 (2)
C17—C16—C21	115.47 (19)	C41—C42—C43	112.0 (2)
N3—C16—C17	128.2 (2)	C38—C43—C42	112.8 (2)
C16—C17—C18	113.6 (2)	C23—C24—H24	121.00
C17—C18—C19	113.8 (2)	C25—C24—H24	121.00
C18—C19—C22	113.2 (3)	C24—C25—H25	120.00
C20—C19—C22	113.5 (3)	C26—C25—H25	120.00
C18—C19—C20	109.6 (2)	C26—C27—H27	121.00
C19—C20—C21	111.3 (2)	C28—C27—H27	121.00
C16—C21—C20	111.7 (2)	C30—C31—H31	120.00
C3—C2—H2	121.00	C32—C31—H31	120.00
C1—C2—H2	121.00	C31—C32—H32	120.00
C4—C3—H3	120.00	C33—C32—H32	120.00
C2—C3—H3	120.00	C32—C33—H33	120.00
C6—C5—H5	122.00	C34—C33—H33	120.00
C4—C5—H5	122.00	C33—C34—H34	120.00
C8—C9—H9	119.00	C35—C34—H34	120.00
C10—C9—H9	119.00	C30—C35—H35	120.00
C9—C10—H10	120.00	C34—C35—H35	120.00
C11—C10—H10	120.00	C38—C39—H39A	109.00
C12—C11—H11	120.00	C38—C39—H39B	109.00
C10—C11—H11	120.00	C40—C39—H39A	109.00
C13—C12—H12	120.00	C40—C39—H39B	109.00
C11—C12—H12	120.00	H39A—C39—H39B	108.00
C12—C13—H13	120.00	C39—C40—H40A	109.00
C8—C13—H13	120.00	C39—C40—H40B	109.00
C18—C17—H17A	109.00	C41—C40—H40A	109.00
H17A—C17—H17B	108.00	C41—C40—H40B	109.00
C16—C17—H17B	109.00	H40A—C40—H40B	108.00
C18—C17—H17B	109.00	C40—C41—H41	107.00
C16—C17—H17A	109.00	C42—C41—H41	107.00
C17—C18—H18A	109.00	C44—C41—H41	107.00
C17—C18—H18B	109.00	C41—C42—H42A	109.00
C19—C18—H18A	109.00	C41—C42—H42B	109.00
H18A—C18—H18B	108.00	C43—C42—H42A	109.00
C19—C18—H18B	109.00	C43—C42—H42B	109.00
C20—C19—H19	107.00	H42A—C42—H42B	108.00
C18—C19—H19	107.00	C38—C43—H43A	109.00
C22—C19—H19	107.00	C38—C43—H43B	109.00
H20A—C20—H20B	108.00	C42—C43—H43A	109.00
C19—C20—H20A	109.00	C42—C43—H43B	109.00
C19—C20—H20B	109.00	H43A—C43—H43B	108.00
C21—C20—H20A	109.00	C41—C44—H44A	109.00
C21—C20—H20B	109.00	C41—C44—H44B	110.00
C16—C21—H21A	109.00	C41—C44—H44C	109.00
C16—C21—H21B	109.00	H44A—C44—H44B	110.00
C20—C21—H21A	109.00	H44A—C44—H44C	109.00
C20—C21—H21B	109.00	H44B—C44—H44C	109.00
			
C1—N1—C14—C15	−179.61 (17)	C17—C16—C21—C20	−47.1 (3)
C14—N1—C1—C2	−177.1 (2)	N3—C16—C17—C18	−138.7 (3)
C14—N1—C1—C6	0.9 (2)	C21—C16—C17—C18	42.9 (3)
C1—N1—C14—C7	−0.6 (2)	C16—C17—C18—C19	−46.9 (3)
C15—N2—N3—C16	−175.2 (2)	C17—C18—C19—C22	−177.5 (2)
N3—N2—C15—O1	6.0 (3)	C17—C18—C19—C20	54.7 (3)
N3—N2—C15—C14	−174.92 (18)	C18—C19—C20—C21	−58.6 (3)
N2—N3—C16—C21	−178.36 (19)	C22—C19—C20—C21	173.7 (3)
N2—N3—C16—C17	3.3 (3)	C19—C20—C21—C16	55.0 (3)
C23—N4—C36—C37	176.82 (18)	C24—C23—C28—C29	−179.1 (2)
C23—N4—C36—C29	−1.0 (2)	N4—C23—C24—C25	−179.6 (2)
C36—N4—C23—C28	0.6 (2)	C28—C23—C24—C25	−0.7 (3)
C36—N4—C23—C24	179.6 (2)	N4—C23—C28—C29	0.0 (2)
C37—N5—N6—C38	178.4 (2)	C24—C23—C28—C27	0.6 (3)
N6—N5—C37—O2	1.3 (3)	N4—C23—C28—C27	179.71 (18)
N6—N5—C37—C36	−179.95 (17)	C23—C24—C25—C26	−0.5 (4)
N5—N6—C38—C39	−1.7 (3)	C24—C25—C26—F2	−177.7 (2)
N5—N6—C38—C43	176.9 (2)	C24—C25—C26—C27	2.0 (4)
C2—C1—C6—C5	−1.9 (3)	C25—C26—C27—C28	−2.1 (3)
N1—C1—C6—C5	179.92 (19)	F2—C26—C27—C28	177.62 (19)
N1—C1—C2—C3	178.9 (2)	C26—C27—C28—C29	−179.6 (2)
C6—C1—C2—C3	1.2 (3)	C26—C27—C28—C23	0.8 (3)
C2—C1—C6—C7	177.4 (2)	C27—C28—C29—C30	−2.5 (4)
N1—C1—C6—C7	−0.8 (2)	C23—C28—C29—C36	−0.6 (2)
C1—C2—C3—C4	0.0 (4)	C27—C28—C29—C36	179.8 (2)
C2—C3—C4—C5	−0.5 (4)	C23—C28—C29—C30	177.10 (18)
C2—C3—C4—F1	178.2 (2)	C28—C29—C36—N4	1.0 (2)
C3—C4—C5—C6	−0.2 (4)	C28—C29—C30—C31	69.5 (3)
F1—C4—C5—C6	−178.8 (2)	C28—C29—C36—C37	−176.4 (2)
C4—C5—C6—C1	1.3 (3)	C30—C29—C36—N4	−176.60 (19)
C4—C5—C6—C7	−177.8 (2)	C36—C29—C30—C35	69.0 (3)
C5—C6—C7—C8	2.9 (4)	C36—C29—C30—C31	−113.3 (2)
C5—C6—C7—C14	179.6 (2)	C30—C29—C36—C37	6.1 (4)
C1—C6—C7—C8	−176.27 (19)	C28—C29—C30—C35	−108.2 (2)
C1—C6—C7—C14	0.4 (2)	C31—C30—C35—C34	−0.7 (3)
C6—C7—C8—C13	−105.2 (3)	C29—C30—C35—C34	177.08 (19)
C14—C7—C8—C13	78.9 (3)	C29—C30—C31—C32	−177.44 (19)
C6—C7—C14—C15	178.9 (2)	C35—C30—C31—C32	0.3 (3)
C6—C7—C14—N1	0.1 (2)	C30—C31—C32—C33	0.2 (3)
C6—C7—C8—C9	76.6 (3)	C31—C32—C33—C34	−0.4 (4)
C8—C7—C14—C15	−4.5 (4)	C32—C33—C34—C35	0.0 (4)
C14—C7—C8—C9	−99.4 (3)	C33—C34—C35—C30	0.5 (3)
C8—C7—C14—N1	176.70 (19)	N4—C36—C37—N5	−164.94 (19)
C9—C8—C13—C12	2.6 (4)	C29—C36—C37—O2	−169.0 (2)
C7—C8—C13—C12	−175.6 (2)	C29—C36—C37—N5	12.2 (3)
C7—C8—C9—C10	174.7 (2)	N4—C36—C37—O2	13.8 (3)
C13—C8—C9—C10	−3.7 (4)	N6—C38—C39—C40	−136.0 (2)
C8—C9—C10—C11	1.8 (4)	C43—C38—C39—C40	45.4 (3)
C9—C10—C11—C12	1.1 (4)	N6—C38—C43—C42	132.9 (2)
C10—C11—C12—C13	−2.1 (4)	C39—C38—C43—C42	−48.3 (3)
C11—C12—C13—C8	0.2 (4)	C38—C39—C40—C41	−49.3 (3)
N1—C14—C15—O1	13.7 (3)	C39—C40—C41—C42	54.3 (3)
C7—C14—C15—N2	15.9 (3)	C39—C40—C41—C44	−179.0 (3)
C7—C14—C15—O1	−165.0 (2)	C40—C41—C42—C43	−55.5 (3)
N1—C14—C15—N2	−165.41 (18)	C44—C41—C42—C43	178.6 (2)
N3—C16—C21—C20	134.3 (2)	C41—C42—C43—C38	53.5 (3)

### Hydrogen-bond geometry (Å, º)

Cg1, Cg2, Cg3, Cg6 and Cg8 are the centroids of the 1*H*-pyrrole and benzene rings of the 1*H*-indole ring system of molecule *A*, the phenyl ring of molecule *A*, the 1*H*-pyrrole ring of the 1*H*-indole ring system of molecule *B* and the phenyl ring of molecule *B*, respectively.

**Table:**

*D*—H···*A*	*D*—H	H···*A*	*D*···*A*	*D*—H···*A*
N1—H1···O2	0.86	2.15	2.895 (2)	145
N4—H4···O1	0.86	2.04	2.811 (2)	149
C17—H17*B*···N2	0.97	2.43	2.811 (3)	103
C39—H39*B*···N5	0.97	2.44	2.814 (3)	102
C17—H17*A*···*Cg*1^i^	0.97	2.66	3.594 (3)	163
C17—H17*B*···*Cg*3	0.97	2.74	3.685 (3)	164
C31—H31···*Cg*6^ii^	0.93	2.87	3.658 (2)	144
C35—H35···*Cg*2^iii^	0.93	2.96	3.627 (3)	130
C39—H39*B*···*Cg*8	0.97	2.72	3.667 (3)	164
C42—H42*A*···*Cg*1^iv^	0.97	2.99	3.848 (3)	148

Symmetry codes: (i) −*x*+1, −*y*+1, −*z*; (ii) −*x*+1, −*y*+2, −*z*+1; (iii) −*x*+1, −*y*+1, −*z*+1; (iv) *x*−1, *y*, *z*.
